# Different phenotypic assessment of depression prevalence in Russian population: DSM-criteria vs HADS

**DOI:** 10.1192/j.eurpsy.2022.1424

**Published:** 2022-09-01

**Authors:** G. Rukavishnikov, A. Rakitko, E. Kasyanov, V. Ilinsky, N. Neznanov, A. Kibitov, G. Mazo

**Affiliations:** 1 Bekhterev National Medical Center for Psychiatry and Neurology, Translational Psychiatry, Saint-Petersburg , Russian Federation; 2 Genotek Ltd., N/a, Moscow, Russian Federation; 3 Bekhterev National Medical Center for Psychiatry and Neurology, Geriatric Psychiatry, Saint-Petersburg , Russian Federation; 4 Serbsky National Medical Research Center on Psychiatry and Addictions, Molecular Genetics Laboratory, Moscow, Russian Federation

**Keywords:** Depression, phenotyping, HADS, Epidemiology

## Abstract

**Introduction:**

Because of different phenotypic approaches, data on depression prevalence is variable and controversial.

**Objectives:**

The aim was to evaluate the prevalence of different depressive phenotypes in the Russian population (DSM criteria based self-report vs HADS questionnaire).

**Methods:**

The data was from the on-line survey of 5116 clients of Genotek Ltd. (males - 50,63%; age - Me=35 (Q1-30;Q3-42)). The survey included questions on sex, age; sel-report adapted major depression DSM-V criteria questionnaire and depression subscale of Hospital Anxiety and Depression Scale.

**Results:**

DSM Major depression phenotype was detected with moderately-high prevalence - 17,67% (N=904). The DSM depression phenotype was more prevalent in women (22,72%) compared to men (12,74%, p<0,001) and in younger individuals (10,18%, p<0,001) compared to older ones (6,16%). HADS-D clinical depression phenotype (score>11) was less prevalent (3,4%) with no significant differences for sex and age. However, the prevalence increased with HADS-D subclinical scores (>8) - 14,97%. HADS-D scores were higher in DSM-depression phenotype individuals compared to ones without DSM phenotype (5,822(3,221) vs. 3,893(2,437), p< 0,001).

**Conclusions:**

Our results showed variable prevalence of depression with different phenotypic approaches. The differences could be associated with the clinical severity of the symptoms and the life-time evaluation in DSM compared to only current symptoms for HADS. Further research is needed to understand the factors affecting the phenotyping approaches and providing the most effective and valid instrument for depression prevalence evaluation. Research is supported by an RSF grant №20-15-00132.

**Disclosure:**

Research is supported by an RSF grant №20-15-00132.

